# Association of Exercise With Vascular Function in Patients With CKD: A Meta-Analysis of Randomized Controlled Trials

**DOI:** 10.3389/fmed.2022.904299

**Published:** 2022-07-06

**Authors:** Huan Wang, Dengpiao Xie, Lisheng Wu, Liangbin Zhao

**Affiliations:** ^1^Hospital of Chengdu University of Traditional Chinese Medicine, Chengdu, China; ^2^Southern Medical University, Guangzhou, China

**Keywords:** chronic kidney disease, dialysis, exercise, vascular function, meta-analysis

## Abstract

**Background and Aim:**

Vascular function is associated with an increased risk of cardiovascular events in patients with chronic kidney disease (CKD). Whether exercise improves vascular function in such patients remains controversial. This study aimed to conduct a meta-analysis on the effect of exercise training on the vascular function of patients with CKD.

**Methods:**

Embase, the Cochrane Central Register of Controlled Trials, and Medline were searched from inception until November 15, 2021. The terms exercise, CKD, dialysis, kidney transplant, and randomized controlled trial (RCT) were searched alone or in combination. RCTs were included when studies compared exercise with active control, usual care, or no intervention, and the studies reported vascular function on patients with CKD.

**Results:**

This meta-analysis included 18 RCTs with 817 patients. Exercise training was significantly associated with decreased pulse wave velocity weighted mean difference (WMD), −0.56; 95% confidence interval (CI), −1.02 to −0.09, *P* = 0.02 and augmentation index (WMD, −3.26; 95% CI, −5.46 to −1.05, *P* = 0.004). It was also significantly associated with improved peak VO2 (WMD, 2.64; 95% CI, 1.94–3.35, *P* < 0.00001), general health (WMD, 7.03; 95% CI, 0.65–13.42, *P* = 0.03), and vitality (WMD, 9.1; 95% CI, 2.50–15.69, *P* = 0.007).

**Conclusions:**

The meta-analysis suggested that exercise training improved vascular function in patients with CKD. An exercise program should be considered as one of the management strategies for vascular dysfunction in patients with CKD. Further studies are needed to demonstrate that exercise training improves cardiovascular diseases in patients with CKD.

## Introduction

The increasing number of patients with chronic kidney disease (CKD) poses a challenge to health care. More than 15% of American adults or 37 million people were estimated to have CKD in 2021 based on data from the Centers for Disease Control and Prevention. Patients with CKD are twice more likely to develop cardiovascular disease (CVD). CVD remains the leading cause of mortality in patients with CKD (1). The increased arterial stiffness is one of the major factors contributing to CVD in such patients. The mechanisms that lead to the arterial disease in CKD include endothelial dysfunction, disorders of nitric oxide metabolism, vascular calcification, and elevation of the levels of pro-inflammatory cytokines (2–4). The complicated mechanisms explain why the treatment focusing on a single risk factor cannot achieve satisfactory outcomes. Previous studies showed that aortic stiffness and carotid stiffness are strongly associated with CVD in patients with CKD (5, 6). Therefore, improving vascular function might bring benefits to these patients.

The safety of exercise training is questioned in patients with CKD because renal perfusion is reduced and proteinuria is more severe in some cases during exercise (7). However, studies also proved the benefits of exercise in patients with CKD. The studies demonstrated that voluntary exercise was an effective therapy to improve endothelial function in rats with CKD (8, 9). In addition, exercise training was shown to improve endothelial function, physical function, inflammatory status, hypertension, nitric oxide availability, and lipid metabolism disorders (10, 11).

Some studies were conducted to assess the effect of exercise on arterial stiffness in patients with CKD. However, most of the studies were non-randomized controlled trials (RCTs), the sample sizes were small, and the results were inconsistent. Therefore, the conclusion was not convincing. Given the lack of high-quality evidence on the effects of exercise on the vascular function of patients with CKD, we conducted a meta-analysis of randomized trials to assess the effect of exercise on the vascular function of such patients.

## Methods

### Methods and Search Strategy

The meta-analysis was performed and reported following Preferred Reporting Items for Systematic Reviews and Meta-analysis (12). The study protocol was registered in the International Prospective Register of Systematic Reviews; registration number: CRD42021283470. Studies were searched in the following databases; Medline, Cochrane Trials, and Embase. The search deadline was November, 12, 2021. The details of the search strategy and terms are presented in Supplementary Table 1. In addition, clinical trial registries and references of similar clinical studies, as well as review articles or systemic reviews on a similar topic, were reviewed to search for potentially relevant studies.

### Data Sources and Study Selection

**Figure 1:**
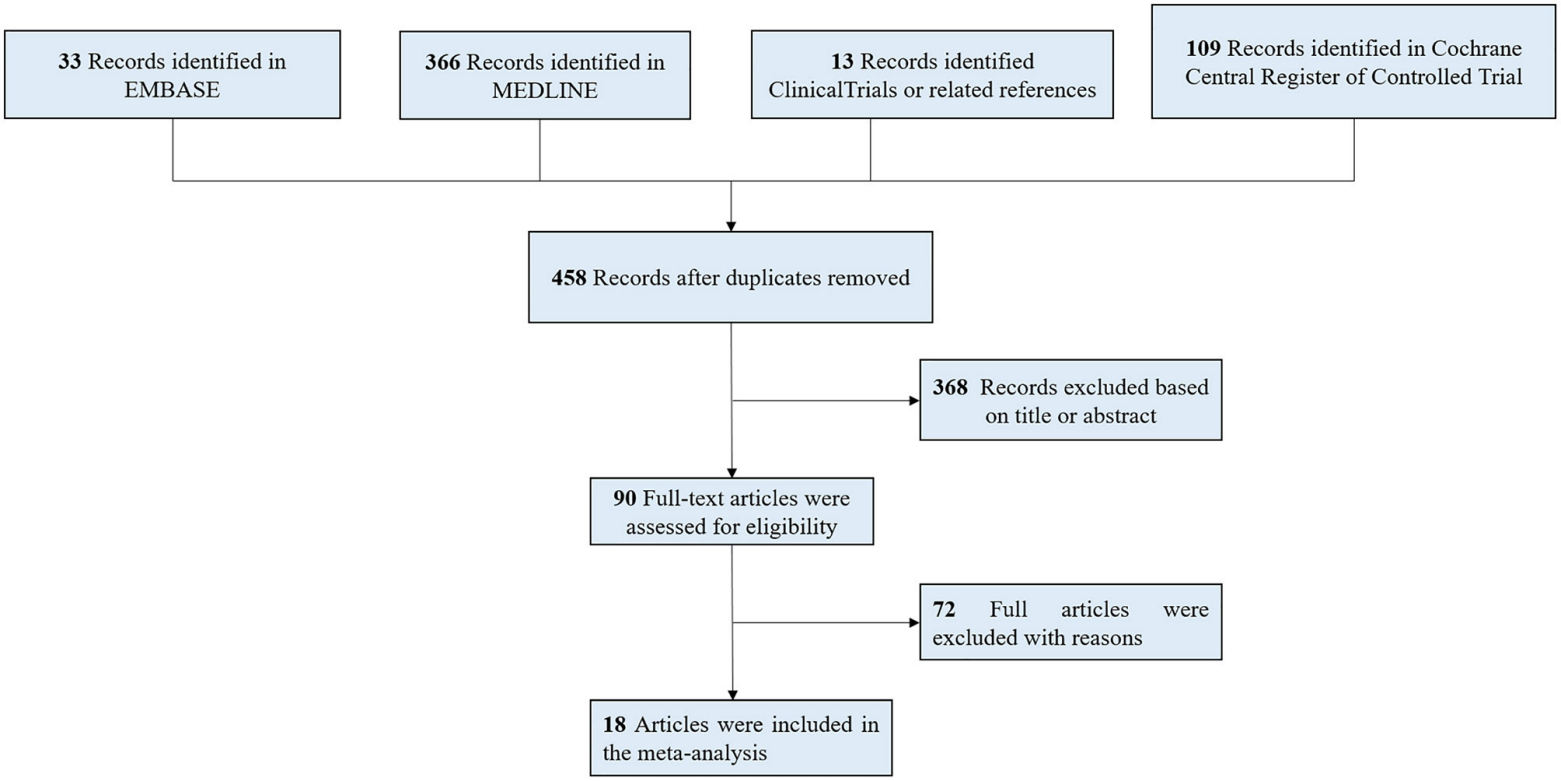
Flow of the search strategy and included studies.

Two independent reviewers (H.W, and D.P.X) evaluated the titles and abstracts and screened the full-text versions of the relevant trials. Disagreements were resolved by consensus between the reviewers, and if necessary, by consulting with other reviewers. The studies were considered for inclusion if they compared exercise with active control, usual care, or no intervention, or they were randomized trials and reported the vascular function in patients with CKD. The flow diagram of study selection is outlined in Figure 1.

### Inclusion and Exclusion Criteria

Patients with CKD, including non-dialysis and dialysis, or patients with kidney transplants were included in the study. The outcome should include indices of vascular function, pulse wave velocity (PWV), or augmentation index. All RCTs that compared exercise with control in managing patients with CKD were also included. This review focused on exercise training. Non-exercise management, such as electrical stimulation and water-based therapy, were excluded. The exercise training included aerobic exercises (such as walking, and cycling on a treadmill, ergometer, or elliptical machine) or resistance training (such as lifting or suppressing the muscle groups). Trials were excluded if they were case reports, comments, letters, or reviews.

### Data Extraction and Quality Assessment

The data on the characteristics of methods, participants, interventions, and outcomes were extracted by two independent reviewers. The Cochrane risk-of-bias tool was used to assess the included trials. It contained the following criteria: selection bias, performance and detection bias, attrition bias, reporting bias, and other sources of bias. Two independent reviewers performed the assessment. The third author resolved the discrepancies. The corresponding authors were responsible for obtaining missing information and unpublished data.

### Data Synthesis and Analysis

The primary outcome was defined as the changes in vascular function, including PWV or augmentation index from baseline to the end of treatment. The changes in peak VO2, health-related quality of life (HRQoL), blood pressure, and C-reactive protein (CRP) level were regarded as the secondary outcomes. If clinical outcomes were measured more than once in a study, we selected the data reported the last time. Data reported as median, interquartile range, 95% confidence interval (CI), or standard error were converted into mean and standard deviation (SD) using the formula (13, 14). We assessed effect size by weighted mean differences (WMDs) for continuous outcomes. The CI was 95%. We assessed heterogeneity with *I*^2^ statistics. An *I*^2^ value <25%, between 25 and 50%, and >75% indicated a low degree of heterogeneity, a moderate degree of heterogeneity, and significant heterogeneity, respectively (15). If the results were not significantly heterogeneous, a fixed-effects model was used. If the results were significantly heterogeneous, a random-effects model was used. The possibility of publication bias for the primary outcome was evaluated using the Egger test and the visual estimate of funnel plot. Sensitivity analyses were conducted by outlier identification and influence analysis using Stata 15. The subgroup analyses were performed based on the duration of the intervention. Exercise training lasting <6 months was defined as short term, while the training lasting ≥6 months was defined as long term. The data were assessed using Review Manager, version 5.3 (Oxford, UK).

## Results

### Literature Selection and Study Characteristics

We identified 521 relevant studies or abstracts by the initial search. After removing 63 duplicates and 368 studies by screening the titles and abstracts, 90 full-text studies were further reviewed in detail. Two articles were considered as the same study for the analyses (16, 17). One study was removed for combining exercise with other lifestyle interventions (18). Finally, 18 studies were included in this meta-analysis.

The summary characteristics of studies included in the meta-analysis are shown in Table 1. All studies were RCTs, enrolling 817 patients. The sample size ranged from 12 to 156 patients, mean sample size of 45 (SD 37). A total of 7 trials (19–24) and NCT03197038 included pre-dialysis patients with CKD, 10 trials included dialysis patients (25–33), and 2 trials included kidney transplant patients (17, 34). Participants received aerobic training in these trials and NCT0319703, resistance training in these trials (17), and both aerobic and resistance trainings in these trials (22, 34). Most of the trials had an exercise frequency of three to four times per week; exercise was performed daily in only one trial (23). The exercise duration varied from 10 to 65 min as can be seen in references (23) and (29), respectively, in each session; only one trial did not report the exercise duration (27). The duration of exercise management was from 2.5 months to 12 months. Two trials contributed to two comparator categories (17, 26). One trial was a cross-over study (25).

**Table 1:** Basic characteristics of subjects and treatments of trials.

**References**	**No. of patients (exercise/control)**	**Type of patient**	**Intervention**		**Duration (months)**
			**Exercise**	**Control**	
Toussaint et al. (25)	19 (9/10)	Dialysis	Bicycling for a minimum of 30 min in each hemodialysis session	Usual care	3
Koh et al. (26)	46 (30/16)	Dialysis	Intradialytic-exercise: Cycling from 15 to 45 min during each dialysis three times per week on the Borg RPE of 12–13. Home-based-exercise: Walking from 15 to 45 min three times per week at Borg RPE of 12–13.	Usual care	6
Mustata et al. (19)	20 (10/10)	CKD3-4	Supervised training included the choice of treadmill, stationary, cycle and elliptical trainer twice per week throughout the study. Home training (walking) was initiated in the 2nd month and progressed over 3 months to a frequency of 3 days/week. Exercise was started at an intensity of 40–60% of peak VO_2_ and duration was up to 60 min at Borg RPE of 12–15	Usual care	12
Kosmadakis et al. (20)	32 (18/14)	CKD4-5	Walking for at least 30 min, five times per week at an RPE of 12–14 and/or achieving the heart rate elicited by this effort level during the tread mill exercise test.	Usual physical activity	6
Riess et al. (34)	31 (16/15)	Kidney transplant	Endurance training was performed on a cycle ergometer and treadmill at 60–80% peak VO_2_ for 30–60 min/session (3 days/week). Strength training was performed at 50% 1RM for 2 sets of 10–15 repetitions (2 days/week)	Usual	3
Headley et al. (21)	46 (25/21)	CKD3	Participants worked at 50–60% peak oxygen uptake using a variety of apparati three times per week.	Usual care	4
Greenwood et al. (22)	18 (8/10)	CKD3-4	Aerobic exercise was performed on recumbent stationary exercise cycles at about RPE of 11 for 40 min three times per week. Resistance training include life or press for upper- and lower- body, starting point of 1–2 sets ×10 repetitions with the aim to increase to 3 sets and 8–10 repetitions three times per week	Usual care	12
Greenwood et al. (17)	46 (26/20)	Kidney transplant	Aerobic exercise was performed on recumbent stationary exercise cycles, a treadmill, and elliptical trainer at PRE of 13–15 for 60 min three times per week. Resistance include lift or press for the upper and lower body muscle groups, starting with 1–2 sets of 10 repetitions with the aim of 3 sets of 8–10 repetitions.	Usual care	3
Van Craenenbroek et al. (23)	40(19/21)	CKD3-4	Four daily cycling sessions of 10 min at a target heat rate calculated as 90% of the heart rate achieved at the anaerobic threshold on baseline testing	Usual care	3
Cooke et al. (27)	20(10/10)	Dialysis	Pedaling exercise to reach 12–16 of RPE for Three times per week.	Usual care	4
Mcgregor et al. (28)	34(18/16)	Dialysis	Cycling was performed for up to 1 h per session to achieve 40–60% oxygen uptake reserve three times per week.	Usual care	2.5
Kirkman et al. (24)	31(16/15)	CKD3-5	Aerobic exercise (cycling, walking/jogging, elliptical) at 60–85% heart rate reserve for 45 min (three times per week)	Usual care	3
Sliva et al. (29)	30 (15/15)	Dialysis	The aerobic training using a cycloergometer lasted 30 min at between 65 and 75% of the maximal heart rate with a Borg scale score around 13 (3 times a week).	Usual care	4
Jeong et al. (30)	67 (29/38)	Dialysis	Cycling 45 min during each dialysis session, and receiving protein supplement.	Usual care and protein supplement	12
Assawasaksakul et al. (31)	12 (6/6)	Dialysis	Cycling for 60 min during each dialysis session with Borg scale score of 13	Usual care	6
Graham-Brown et al. (33)	130(65/65)	Dialysis	Cycling for 30 min with 12–14 of RPE three times per week during dialysis.	Usual care	6
Greenwood et al. (32)	156 (78/78)	Dialysis	Cycling start from 21 min and progressing to 40 min per dialysis session.	Usual care	6
NCT03197038	39(22/17)	CKD	Participants exercised (a brisk walk) at home, for 30–60 min, 3 times per week	Usual care	6

*CKD, Chronic kidney disease*.

### Risk-of-Bias Assessment

All studies were randomized trials included in this meta-analysis. Among these studies, 12 trials (17, 19, 21–26, 28, 31, 32, 34) reported the concrete randomization methods. The performance bias was considered as high risk in all trials because it was impossible to blind the participants and researches for the exercise training. The intention-to-treat approach was employed in these trials (19, 29, 33). The risk-of-bias assessments are presented in Figure 2 in the supplement.

**Figure 2:**
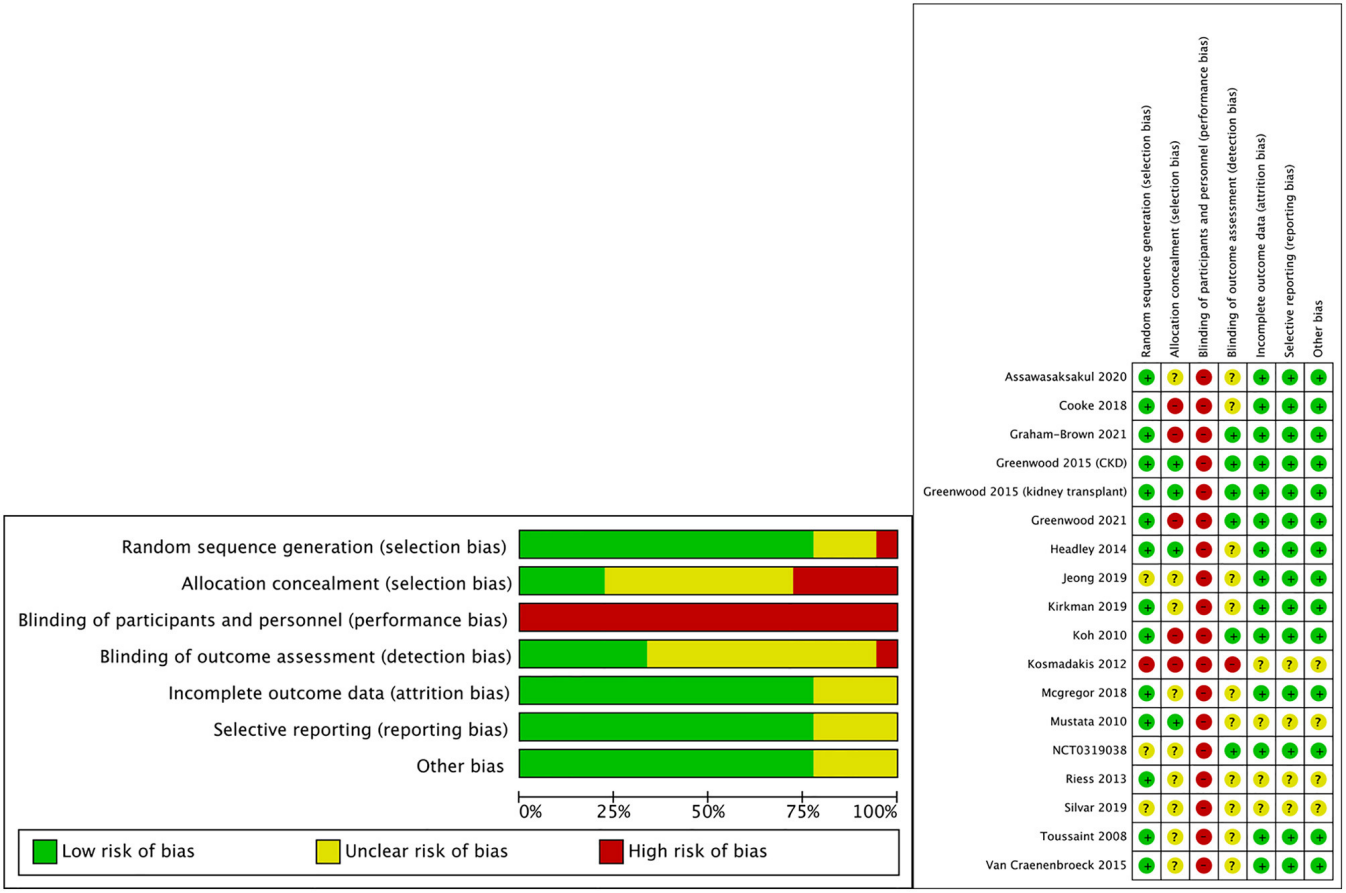
Risk of bias graph of included studies.

### Primary Outcome: PWV and Augmentation Index

**Figure 3:**
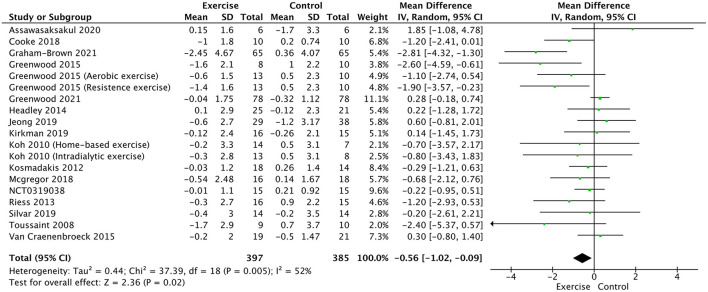
Forest plot of exercise training on PWV.

A total of 17 trials (17, 20–34) and NCT03197038 were included in the meta-analysis for PWV between the two groups. The result showed that exercise training significantly decreased PWV in patients with CKD (WMD, −0.56; 95% CI, −1.02 to −0.09, *P* = 0.02, without significant heterogeneity; *P* = 0.005, *I*^2^ = 52%, Figure 3).

**Figure 4:**
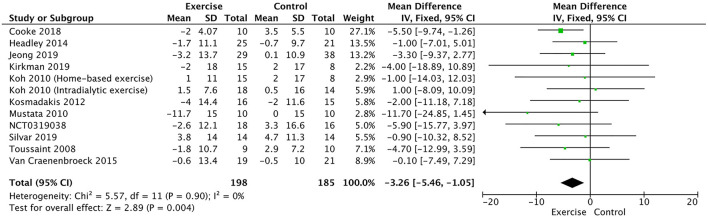
Forest plot of exercise training on augmentation index.

The augmentation index was measured in 11 trials (19–21, 23–27, 29, 30) and NCT03197038. The pooled result showed that exercise training significantly decreased the augmentation index (WMD, −3.26; 95% CI, −5.46 to −1.05, *P* = 0.004; without heterogeneity: *P* = 0.90, *I*^2^ = 0%, Figure 4).

### Secondary Outcome: Peak VO2, CRP, Blood Pressure, and HRQoL

**Figure 5:**
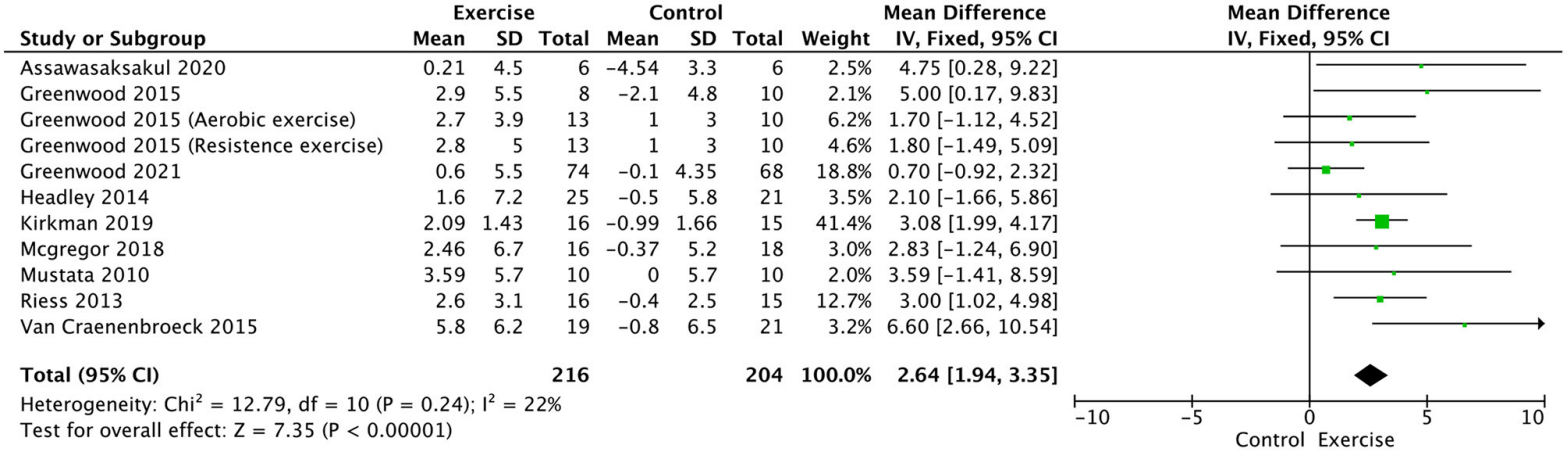
Forest plot of exercise training on peak VO2.

Peak VO2 was compared in 10 trials (17, 19, 21–24, 28, 31, 32, 34). It significantly increased in the exercise training group compared with the usual control group (WMD, 2.64; 95% CI, 1.94–3.35, *P* < 0.00001; without heterogeneity: *P* = 0.24, *I*^2^ = 22%, Figure 5).

The blood pressure was compared in 11 trials (17, 21–28, 30, 33). the exercise training had no effect on either systolic blood pressure (WMD, −0.70; 95% CI, −4.28 to 2.87, *P* = 0.70; without heterogeneity: *P* = 0.87, *I*^2^ = 0%, Supplementary Figure 1) or diastolic blood pressure (WMD, −0.55; 95% CI, −2.83 to 1.74, *P* = 0.64; without heterogeneity: *P* = 0.84, *I*^2^ = 0%, Supplementary Figure 2).

CRP was compared in seven trials (17, 21, 23, 25, 29–31). The exercise training had no effect on the levels of CRP (WMD, −0.09; 95% CI, −0.26 to 0.09, *P* = 0.33; without heterogeneity: *P* = 0.57, *I*^2^ = 0%, Supplementary Figure 3).

HRQoL was compared in four trials (19, 21, 23, 26), including vitality, general health, social function pain, and mental health. No significant difference in mental health was found between the training and control groups (WMD, 1.09; 95% CI, −4.21 to 6.4, *P* = 0.69; without heterogeneity: *P* = 0.31, *I*^2^ = 16%, Supplementary Figure 4), social function (WMD, 4.08; 95% CI, −2.52 to 10.69, *P* = 0.23; without heterogeneity: *P* = 0.88, *I*^2^ = 0%, Supplementary Figure 5). However, exercising training improved general health (WMD, 7.03; 95% CI, 0.65–13.42, *P* = 0.03; without heterogeneity: *P* = 0.75, *I*^2^ = 0%, Supplementary Figure 6) and vitality (WMD, 9.1; 95% CI, 2.50–15.69, *P* = 0.007; without heterogeneity: *P* = 0.91, *I*^2^ = 0%, Supplementary Figure 7).

### Subgroup Analysis

The subgroup analysis revealed that PWV was significantly lower in patients with short-term exercise training and without heterogeneity (Supplementary Figure 8). However, no difference was observed between the long-term exercise training group and the control group in patients with significant heterogeneity (Supplementary Figure 8).

### Adverse Events

Among the studies, nine trials (17, 19, 22, 24–28, 31, 34) reported no adverse events with exercise. Two trials (31, 32) reported no difference in adverse events between exercise training groups and control group. One trial (33) reported that the exercise training groups had more adverse events than the control group; the adverse events were judged to have no relationship with exercise. The study by Graham-Brown et al. (33) reported two deaths in each group, while Greenwood et al. (32) reported three deaths in the exercise training group and four deaths in the control group.

### Sensitivity Analysis and Publication Bias

**Figure 6:**
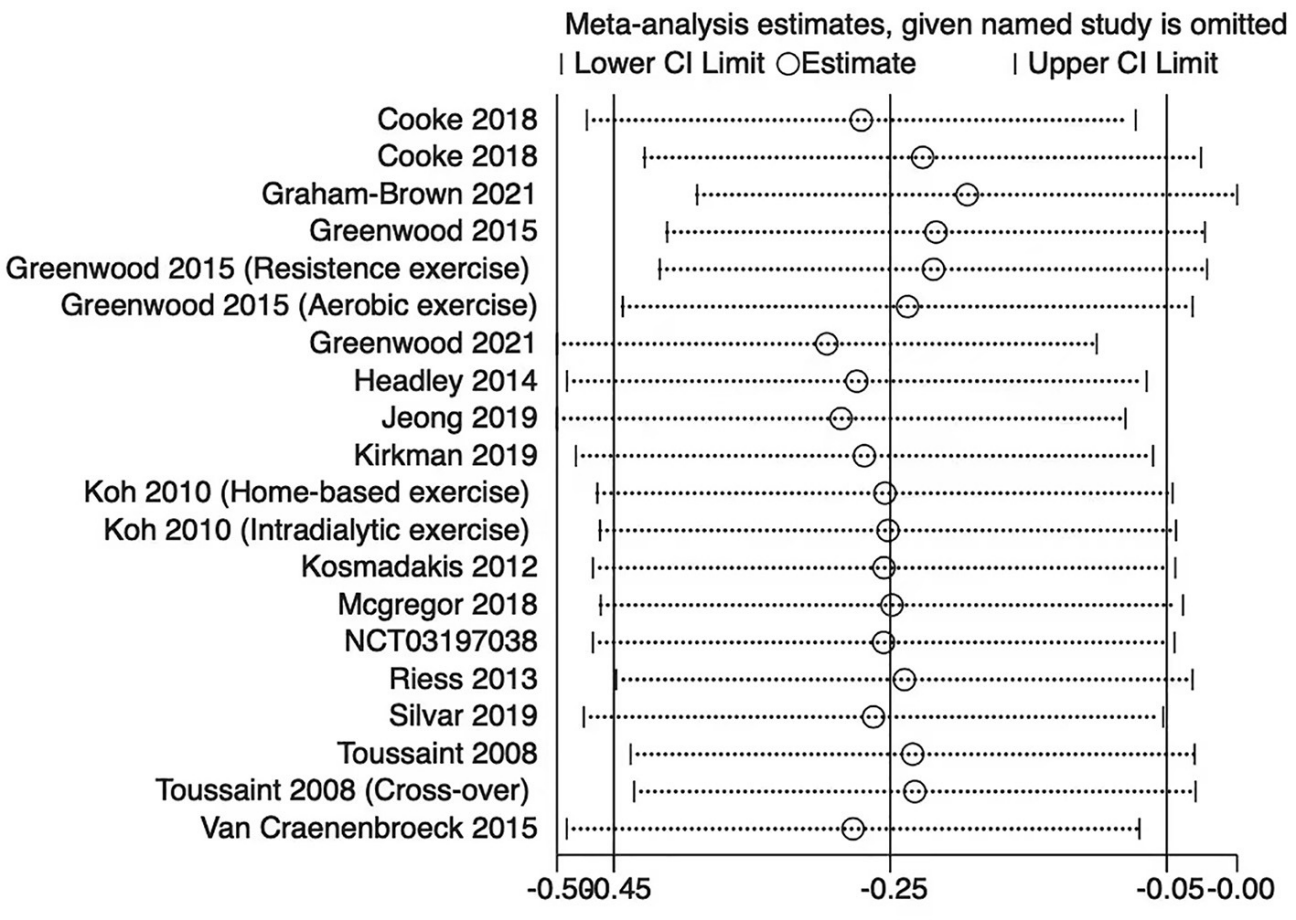
Leave-one-out forest plot for the PWV.

**Figure 7:**
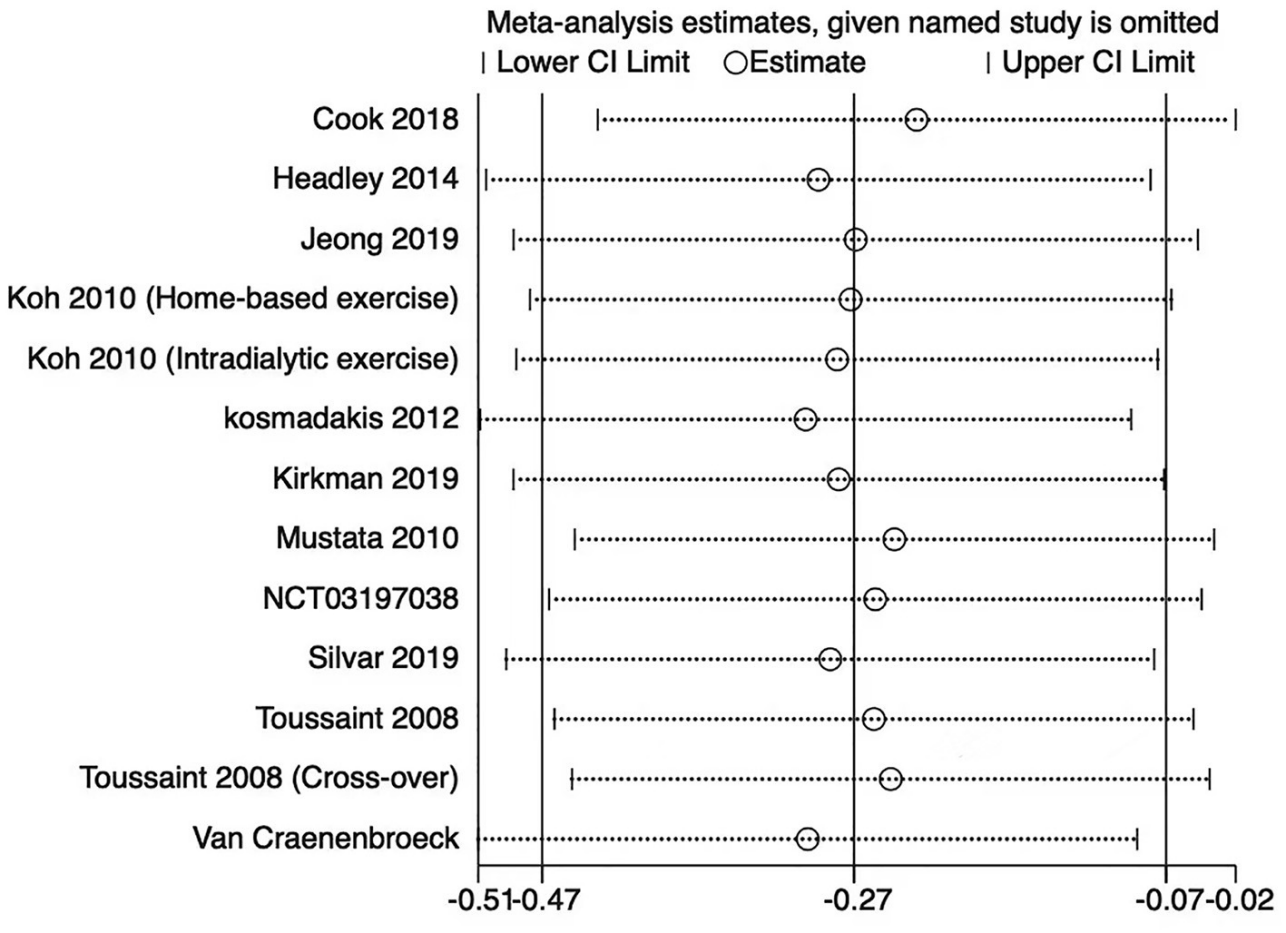
Leave-one-out forest plot for the augmentation index.

The sensitivity analysis was performed by leave-one-out analysis in the primary outcomes. The leave-one-out analysis showed that the pooled result and heterogeneity had no significant change in PWV (Figure 6) and augmentation index (Figure 7).

**Figure 8:**
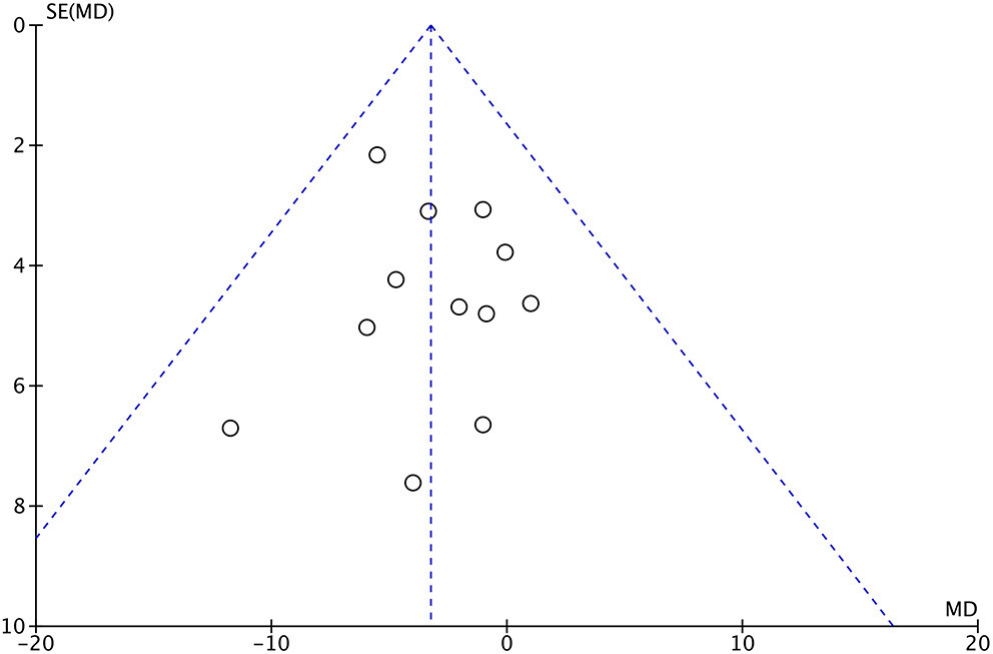
Funnel plot for augmentation index.

**Figure 9:**
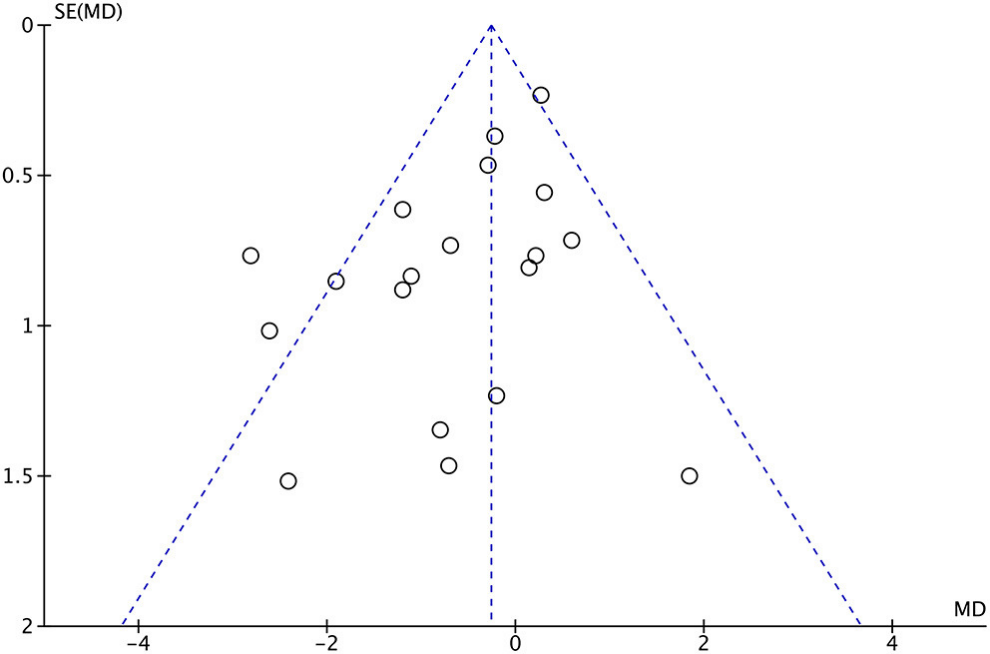
Funnel plot for PWV.

The funnel plot analysis showed the symmetry in Figure 8, and the Egger test (*P* > 0.05) did not detect the significant publication bias for the augmentation index. However, the funnel plot analysis showed some asymmetry in Figure 9, and the Egger test (*P* < 0.05) detected the publication bias for PWV.

## Discussion

This meta-analysis involved 18 trials with 817 patients and showed that exercise training was significantly associated with reduced arterial stiffness evidenced by decreased PWV and augmentation index. In addition, exercise training was associated with improved peak VO2, general health, and vitality. However, no association of exercise training with improved CRP, mental health, and social function was found in this meta-analysis. Finally, this meta-analysis found no association between exercise training and adverse events.

Vascular stiffness is common in patients with CKD, and worsens as kidney function declines (35). The calcification of arteries is associated with vascular stiffness, which is an independent risk factor for CVD (36). PWV is the most widely used parameter for assessing arterial stiffness; it has become a useful method for diagnosis, risk stratification, and prognosis of cardiovascular diseases (37). It has been demonstrated to be associated with cardiovascular and all-cause mortality in patients with end-stage renal disease (ESRD) (38). Blacher et al. demonstrated an increase of 1 m/s in PWV in patients with ESRD, while the all-cause mortality increased by 1.39 times (6). The augmentation index is another useful tool to reflect the arterial stiffness and predict cardiovascular outcomes (39, 40). London et al. provided direct evidence that an increased effect of augmentation index was a predictor of all-cause and cardiovascular mortality in patients with CKD (41). Therefore, PWV and augmentation index were considered as the primary outcomes in this meta-analysis. Although PWV and augmentation index are independent predictors of cardiovascular events, they are affected by different factors. PWV might be affected by blood pressure, distensibility of the arterial wall, and peripheral vascular resistance, while augmentation index might be affected by ventricular ejection and heart rate (42, 43). This meta-analysis had similar results, showing that exercise training reduced PWV and augmentation index, which made the conclusion of this meta-analysis more convincing. In these non-RCTs, the results showed that exercise improved vascular function, as evidenced by improved flow-mediated dilation (44, 45), which were consistent with our results.

Several meta-analyses assessed the effect of exercise on patients with CKD. However, these studies focused on aerobic capacity, muscular function, or health-related quality of life. No meta-analysis study focused on the effect of exercise on arterial stiffness in patients with CKD. In 2014, a meta-analysis included 928 patients with CKD. It found that exercise improved aerobic capacity, muscular function, and health-related quality of life (46). In 2019, similar meta-analyses found that aerobic exercise improved aerobic capacity, exercise duration, and health-related quality of life (47, 48) in patients with CKD and those undergoing hemodialysis. In 2019, a meta-analysis helped reinforce our findings. The review found that exercise improved PWV; however, only two trials were included (49).

Considering limited data on patients with CKD in terms of the benefits and risks of exercise interventions, the Kidney Disease Improving Global Outcomes (KDIGO) guideline followed the guideline of the American Heart Association (AHA), in which exercise was suggested for preventing cardiovascular diseases in patients with CKD. This meta-analysis provided a rationale for the KDIGO and AHA recommendation of exercise in the management strategy for cardiovascular diseases in patients with CKD. These findings indicated that three to four times of aerobic exercise was appropriate for such patients. However, the optimal duration of exercise each time and the beginning exercise of the CKD stage to achieve maximal benefits remain unknown. Further trials are needed to examine the suitable duration and type of exercise with a personalized condition for patients with CKD who are more likely to adhere and achieve benefit.

### Limitations

This review had some limitations. First, we observed moderate levels of heterogeneity in PWV using *I*^2^ statistics. We further conducted subgroups analysis to reduce heterogeneity based on the duration of exercise training. The heterogeneity of PWV decreased significantly in short-term exercise training; however, the heterogeneity of PWV was even higher in long-term exercise training, which might be the main reason for the reverse outcome. We were unable to use more meaningful subgroups to reduce heterogeneity for PWV. Second, although we conducted a comprehensive search of clinical trial registries and literature to reduce the risk of missing any study, an asymmetry funnel plot and Egger test detected publication bias. The potential sources of publication bias might include selective outcome reporting, English language bias, and differences in methodological quality among trials (50). Third, we did not evaluate the important covariates, such as the association of age with the primary outcomes, due to the low number of trials to conduct a convincing meta-regression. Fourth, this meta-analysis found that exercise training reduced arterial stiffness. However, we did not compare the effects of aerobic exercise training and resistance exercise training on these patients due to the low number of trials.

## Conclusions

The meta-analysis suggested that exercise training improved vascular function in patients with CKD. An exercise program should be considered as one of the management strategies for vascular dysfunction in patients with CKD. Further studies are needed to demonstrate that exercise training improves CVD in patients with CKD.

## Data Availability Statement

The original contributions presented in the study are included in the article/Supplementary Material, further inquiries can be directed to the corresponding author/s.

## Author Contributions

HW, DX, and LZ contributed to the collection of data, bias assessment, data analysis, and manuscript writing. LW and LZ contributed to bias assessment and data extraction. LZ and DX contributed to the design of the study. All authors contributed to the article and approved the submitted version.

## Funding

This work was supported by the Sichuan Administration of Traditional Chinese Medicine (Grant no. 2021MS123) and Special Project for the Development of Traditional Chinese Medicine (Grant no. CYW2022046).

## Conflict of Interest

The authors declare that the research was conducted in the absence of any commercial or financial relationships that could be construed as a potential conflict of interest.

## Publisher's Note

All claims expressed in this article are solely those of the authors and do not necessarily represent those of their affiliated organizations, or those of the publisher, the editors and the reviewers. Any product that may be evaluated in this article, or claim that may be made by its manufacturer, is not guaranteed or endorsed by the publisher.

## Abbreviations

RCTs: randomized controlled trials
CKD: chronic kidney disease
PWV: pulse wave velocity
WMD: weighted mean difference
CI: confidence interval
CVD: cardiovascular disease
PRISMA: Systematic Reviews and Meta-analysis
HRQoL: health-related quality of life
CRP: C-reactive protein
SD: standard deviation
WMD: weighted mean difference.
