# Supplementary material for: Association of Exercise With Vascular Function in Patients With CKD: A Meta-Analysis of Randomized Controlled Trials
Source: Front Med (Lausanne). 2022 Jul 6;9:904299. doi: 10.3389/fmed.2022.904299 (PMC9299368; doi:10.3389/fmed.2022.904299)

**Association of exercise with vascular function in patients with CKD: a meta-analysis of randomized controlled trials**

Supplementary Online Content

eTable 1. Search strategy.

eFigure 1: Forest plot for systolic blood pressure.

eFigure 2: Forest plot for diastolic blood pressure.

eFigure 3: Forest plot for CRP.

eFigure 4: Forest plot for mental health.

eFigure 5: Forest plot for social function.

eFigure 6: Forest plot for general health.

eFigure 7: Forest plot for vitality.

eFigure 8: Subgroup analysis for PWV based on duration of exercise training.

eTable 1. Search strategy.

| MEDLINE(R) | | |
| --- | --- | --- |
| 1 | "Exercise"[Mesh] | **217789** |
| 2 | (((((((((((((((((((((((((Exercises[Title/Abstract]) ) OR (Physical Activity[Title/Abstract])) OR (Activities, Physical[Title/Abstract])) OR (Activity, Physical[Title/Abstract])) OR (Physical Activities[Title/Abstract])) OR (Exercise, Physical[Title/Abstract])) OR (Exercises, Physical[Title/Abstract])) OR (Physical Exercise[Title/Abstract])) OR (Physical Exercises[Title/Abstract])) OR (Acute Exercise[Title/Abstract])) OR (Acute Exercises[Title/Abstract])) OR (Exercise, Acute[Title/Abstract])) OR (Exercises, Acute[Title/Abstract])) OR (Exercise, Isometric[Title/Abstract])) OR (Exercises, Isometric[Title/Abstract])) OR (Isometric Exercises[Title/Abstract])) OR (Isometric Exercise[Title/Abstract])) OR (Exercise, Aerobic[Title/Abstract])) OR (Aerobic Exercise[Title/Abstract])) OR (Aerobic Exercises[Title/Abstract])) OR (Exercises, Aerobic[Title/Abstract])) OR (Exercise Training[Title/Abstract])) OR (Exercise Trainings[Title/Abstract])) OR (Training, Exercise[Title/Abstract])) OR (Trainings, Exercise[Title/Abstract]) | **122615** |
| 3 | "Renal Insufficiency, Chronic"[Mesh] | **122615** |
| 4 | ((((((((((((((((((((((((((((Chronic Renal Insufficiencies[Title/Abstract]) OR (Renal Insufficiencies, Chronic[Title/Abstract])) OR (Chronic Renal Insufficiency[Title/Abstract])) OR (Kidney Insufficiency, Chronic[Title/Abstract])) OR (Chronic Kidney Insufficiency[Title/Abstract])) OR (Chronic Kidney Insufficiencies[Title/Abstract])) OR (Kidney Insufficiencies, Chronic[Title/Abstract])) OR (Chronic Kidney Diseases[Title/Abstract])) OR (Chronic Kidney Disease[Title/Abstract])) OR (Disease, Chronic Kidney[Title/Abstract])) OR (Diseases, Chronic Kidney[Title/Abstract])) OR (Kidney Disease, Chronic[Title/Abstract])) OR (Kidney Diseases, Chronic[Title/Abstract])) OR (Chronic Renal Diseases[Title/Abstract])) OR (Chronic Renal Disease[Title/Abstract])) OR (Disease, Chronic Renal[Title/Abstract])) OR (Diseases, Chronic Renal[Title/Abstract])) OR (Renal Disease, Chronic[Title/Abstract])) OR (Renal Diseases, Chronic[Title/Abstract])) OR (Renal dialysis[Title/Abstract])) OR (Dialyses, Renal[Title/Abstract])) OR (Renal Dialyses[Title/Abstract])) OR (Dialysis, Renal[Title/Abstract])) OR (Hemodialysis[Title/Abstract])) OR (Hemodialyses[Title/Abstract])) OR (Dialysis, Extracorporeal[Title/Abstract])) OR (Dialyses, Extracorporeal[Title/Abstract])) OR (Extracorporeal Dialyses[Title/Abstract])) OR (Extracorporeal Dialysis[Title/Abstract]) | **88954** |
| 5 | (transplantation[Title/Abstract]) OR (transplant[Title/Abstract]) | [**457,238**](https://pubmed.ncbi.nlm.nih.gov/?term=%28transplantation%5BTitle%2FAbstract%5D%29+OR+%28transplant%5BTitle%2FAbstract%5D%29&sort=) |
| 6 | Vascular Stiffness"[Mesh] | **7028** |
| 7 | (vascular[Title/Abstract]) OR (arterial[Title/Abstract]) OR ((Vascular Stiffness[MeSH Terms]) OR (((((((((Vascular function[Title/Abstract]) OR (Stiffness, Vascular[Title/Abstract])) OR (Vascular Stiffnesses[Title/Abstract])) OR (Arterial Stiffness[Title/Abstract])) OR (Arterial Stiffnesses[Title/Abstract])) OR (Stiffness, Arterial[Title/Abstract])) OR (Aortic Stiffness[Title/Abstract])) OR (Aortic Stiffnesses[Title/Abstract])) OR (Stiffness, Aortic[Title/Abstract]))) | [**931,865**](https://pubmed.ncbi.nlm.nih.gov/?term=%28%22Vascular+Stiffness%22%5BMesh%5D%29+OR+%28%28vascular%5BTitle%2FAbstract%5D%29+OR+%28arterial%5BTitle%2FAbstract%5D%29+OR+%28%28Vascular+Stiffness%5BMeSH+Terms%5D%29+OR+%28%28%28%28%28%28%28%28%28Vascular+function%5BTitle%2FAbstract%5D%29+OR+%28Stiffness%2C+Vascular%5BTitle%2FAbstract%5D%29%29+OR+%28Vascular+Stiffnesses%5BTitle%2FAbstract%5D%29%29+OR+%28Arterial+Stiffness%5BTitle%2FAbstract%5D%29%29+OR+%28Arterial+Stiffnesses%5BTitle%2FAbstract%5D%29%29+OR+%28Stiffness%2C+Arterial%5BTitle%2FAbstract%5D%29%29+OR+%28Aortic+Stiffness%5BTitle%2FAbstract%5D%29%29+OR+%28Aortic+Stiffnesses%5BTitle%2FAbstract%5D%29%29+OR+%28Stiffness%2C+Aortic%5BTitle%2FAbstract%5D%29%29%29%29&sort=) |
| 8 | (1 OR 2) and (3 OR 4 OR 5) and (6 OR 7) | 407 |
| 9 | Randomized controlled trial | **724413** |
| 10 | Controlled clinical trial | **789828** |
| 11 | Randomized | **1312467** |
| 12 | Placebo | **246562** |
| 13 | Randomly | **367609** |
| 14 | Human | 20542615 |
| 15 | 9 OR 10 OR 11 OR 12 OR 13 OR 14 | 20941145 |
| 16 | 7 OR 15 | 366 |
| Cochrane Central Register of Controlled Trials | | |
| 1 | MeSH (exercise) | 26655 |
| 2 | (Exercises):ab,ti,kw OR (Physical Activity):ab,ti,kw OR (Activities, Physical):ab,ti,kw OR (Activity, Physical):ab,ti,kw (Physical Activities):ab,ti,kw OR (Exercise, Physical):ab,ti,kw OR (Exercises, Physical):ab,ti,kw OR (Physical Exercise):ab,ti,kw OR (Physical Exercises):ab,ti,kw OR (Acute Exercise):ab,ti,kw OR (Acute Exercises):ab,ti,kw OR (Exercise, Acute):ab,ti,kw OR (Exercises, Acute):ab,ti,kw OR (Exercise, Isometric):ab,ti,kw OR (Exercises, Isometric):ab,ti,kw OR (Isometric Exercises):ab,ti,kw OR (Isometric Exercise):ab,ti,kw OR (Exercise, Aerobic):ab,ti,kw OR (Aerobic Exercise):ab,ti,kw OR (Aerobic Exercises):ab,ti,kw OR (Exercises, Aerobic):ab,ti,kw OR (Exercise Training):ab,ti,kw OR (Exercise Trainings):ab,ti,kw OR (Training, Exercise):ab,ti,kw OR (Trainings, Exercise):ab,ti,kw | 103311 |
| 3 | MeSH (kidney failure, chronic) | 4804 |
| 4 | (Chronic Renal Insufficiencies):ti,ab,kw OR (Chronic Renal Insufficiencies):ti,ab,kw OR (Renal Insufficiencies, Chronic):ti,ab,kw OR (Chronic Renal Insufficiency):ti,ab,kw OR (Kidney Insufficiency, Chronic):ti,ab,kw OR (Chronic Kidney Insufficiency):ti,ab,kw OR (Chronic Kidney Insufficiencies):ti,ab,kw (Kidney Insufficiencies, Chronic):ti,ab,kw OR (Chronic Kidney Diseases):ti,ab,kw OR (Chronic Kidney Disease):ti,ab,kw OR (Disease, Chronic Kidney):ti,ab,kw OR (Diseases, Chronic Kidney):ti,ab,kw OR (Kidney Disease, Chronic):ti,ab,kw OR (Kidney Diseases, Chronic):ti,ab,kw OR (Chronic Renal Diseases):ti,ab,kw OR (Chronic Renal Disease):ti,ab,kw OR (Disease, Chronic Renal):ti,ab,kw OR (Diseases, Chronic Renal):ti,ab,kw OR (Renal Disease, Chronic):ti,ab,kw OR (Renal Diseases, Chronic):ti,ab,kw OR (Renal dialysis):ti,ab,kw OR (Dialyses, Renal):ti,ab,kw OR (Renal Dialyses):ti,ab,kw OR (Dialysis, Renal):ti,ab,kw OR (Hemodialysis):ti,ab,kw OR (Hemodialyses):ti,ab,kw OR (Dialysis, Extracorporeal):ti,ab,kw OR (Dialysis, Extracorporeal):ti,ab,kw OR (Dialysis, Extracorporeal):ti,ab,kw OR (Dialyses, Extracorporeal):ti,ab,kw OR (Extracorporeal Dialyses):ti,ab,kw OR (Extracorporeal Dialysis):ti,ab,kw OR (transplation):ti,ab,kw OR (transplat):ti,ab,kw | 27941 |
| 5 | Mesh (Vascular Stiffness) | 597 |
| 6 | (Stiffness, Vascular):ti,ab,kw OR (Vascular Stiffnesses):ti,ab,kw OR (Arterial Stiffness):ti,ab,kw OR (Arterial Stiffnesses):ti,ab,kw OR (Stiffness, Arterial):ti,ab,kw OR (Aortic Stiffness):ti,ab,kw OR (Aortic Stiffnesses):ti,ab,kw OR (Stiffness, Aortic):ti,ab,kw OR (vascular function):ti,ab,kw | 14041 |
| 7 | (#1 or #2) and (#3 or #4) and (#5 or #6) | 109 |
| Embase | | |
| 1 | 'exercise'/exp | 298990 |
| 2 | 'Exercises':ab,ti OR 'Physical Activity':ab,ti OR 'Activities, Physical':ab,ti OR 'Activity, Physical':ab,ti OR 'Physical Activities':ab,ti OR 'Exercise, Physical':ab,ti OR 'Exercises, Physical':ab,ti OR 'Physical Exercise':ab,ti OR 'Physical Exercises':ab,ti OR 'Acute Exercise':ab,ti OR 'Acute Exercises':ab,ti OR 'Exercise, Acute':ab,ti OR 'Exercises, Acute':ab,ti OR 'Exercise, Isometric':ab,ti OR 'Exercises, Isometric':ab,ti OR 'Isometric Exercises':ab,ti OR 'Isometric Exercise':ab,ti OR 'Exercise, Aerobic':ab,ti OR 'Aerobic Exercise':ab,ti OR 'Aerobic Exercises':ab,ti OR 'Exercises, Aerobic':ab,ti OR 'Exercise Training':ab,ti OR 'Exercise Trainings':ab,ti OR 'Training, Exercise':ab,ti OR 'Trainings, Exercise':ab,ti | 282020 |
| 3 | 'chronic kidney failure'/exp | 171479 |
| 4 | 'renal insufficiency, chronic':ab,ti OR 'chronic renal insufficiencies':ab,ti OR 'renal insufficiencies, chronic':ab,ti OR 'chronic renal insufficiency':ab,ti OR 'kidney insufficiency, chronic':ab,ti OR 'chronic kidney insufficiency':ab,ti OR 'chronic kidney insufficiencies':ab,ti OR 'kidney insufficiencies, chronic':ab,ti OR 'chronic kidney diseases':ab,ti OR 'chronic kidney disease':ab,ti OR 'disease, chronic kidney':ab,ti OR 'diseases, chronic kidney':ab,ti OR 'kidney disease, chronic':ab,ti OR 'kidney diseases, chronic':ab,ti OR 'chronic renal diseases':ab,ti OR 'chronic renal disease':ab,ti OR 'disease, chronic renal':ab,ti OR 'diseases, chronic renal':ab,ti OR 'renal disease, chronic':ab,ti OR 'renal diseases, chronic':ab,ti OR 'renal dialysis':ab,ti OR 'dialyses, renal':ab,ti OR 'renal dialyses':ab,ti OR 'dialysis, renal':ab,ti OR 'hemodialysis':ab,ti OR 'hemodialyses':ab,ti OR 'dialysis, extracorporeal':ab,ti OR 'dialyses, extracorporeal':ab,ti OR 'extracorporeal dialyses':ab,ti OR 'extracorporeal dialysis':ab,ti OR 'transplantation':ab,ti OR 'transplant':ab,ti | 813069 |
| 5 | 'arterial stiffness'/exp | 24237 |
| 6 | 'vascular function':ab,ti OR 'vascular stiffness':ab,ti OR 'stiffness, vascular':ab,ti OR 'vascular stiffnesses':ab,ti OR 'arterial stiffness':ab,ti OR 'arterial stiffnesses':ab,ti OR 'stiffness, arterial':ab,ti OR 'aortic stiffness':ab,ti OR 'aortic stiffnesses':ab,ti OR 'stiffness, aortic':ab,ti | 32926 |
| 7 | 'random*':ab,ti OR 'placebo*':ab,ti OR 'double-blind':ab,ti OR 'ramdomly':ab,ti OR 'randomized controlled trial':ab,ti | 1856392 |
| 8 | (#1 or #2) and (#3 or #4) and (#5 or #6) and #7 | 33 |


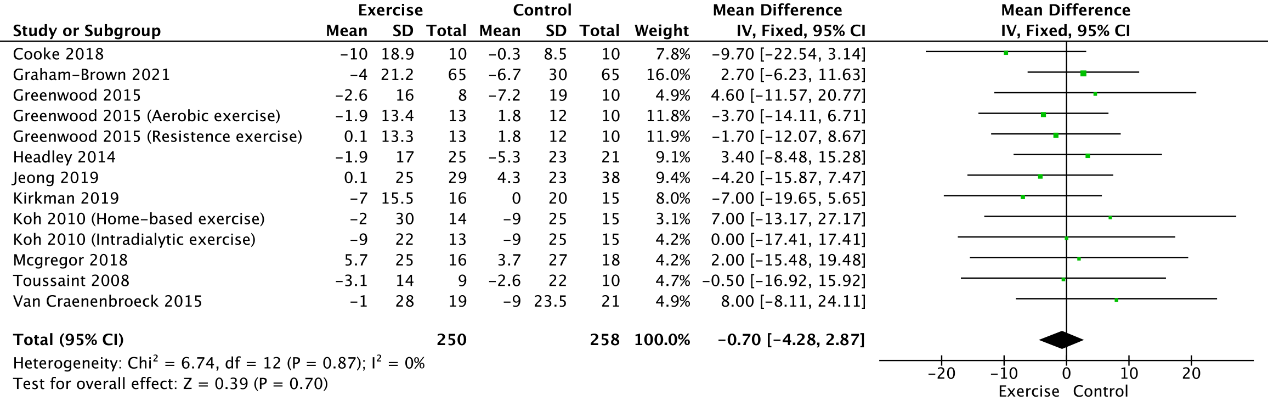
eFigure 1: Forest plot for systolic blood pressure.


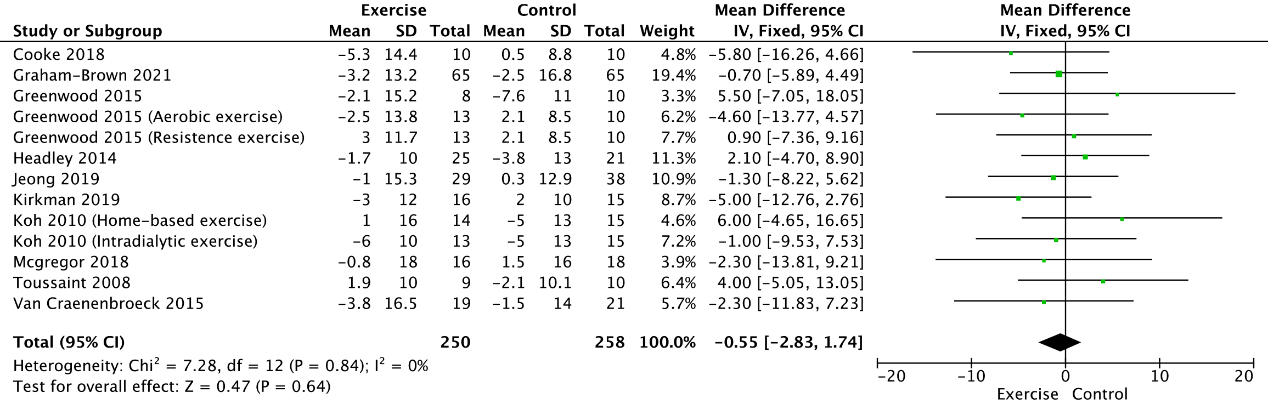
eFigure 2: Forest plot for diastolic blood pressure.


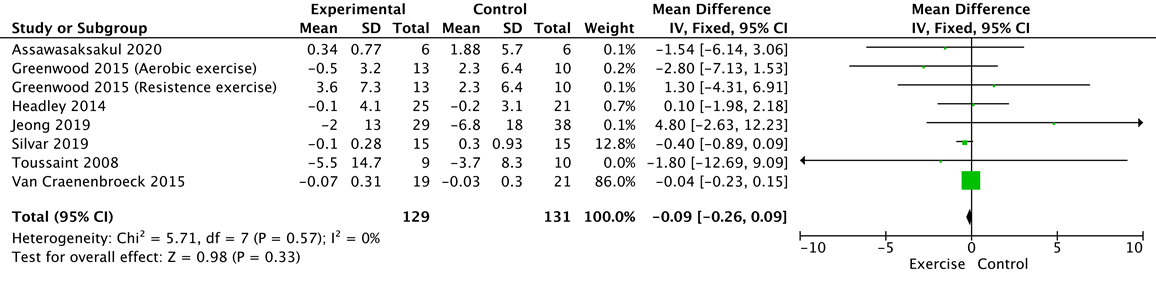
eFigure 3: Forest plot for CRP.

eFigure 4: Forest plot for mental health.


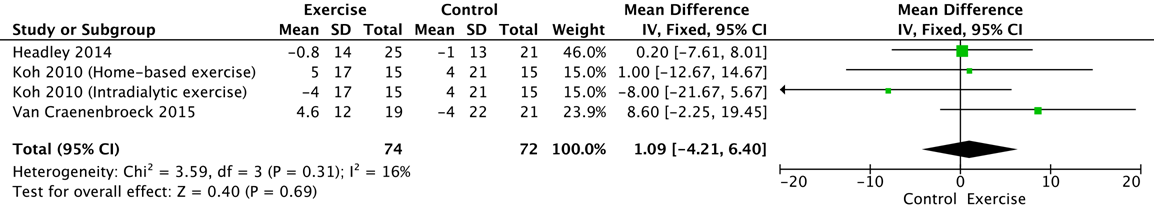


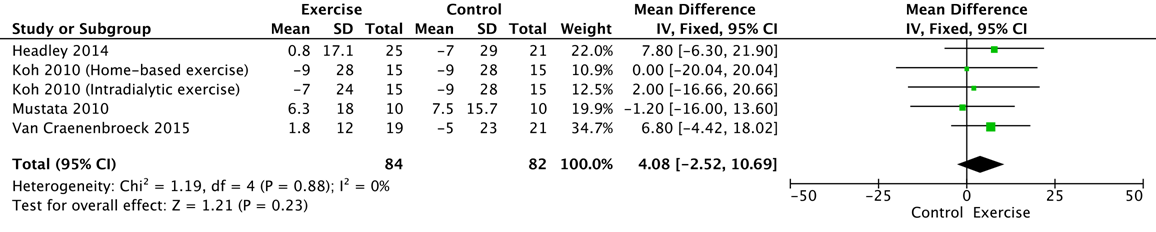
eFigure 5: Forest plot for social function.


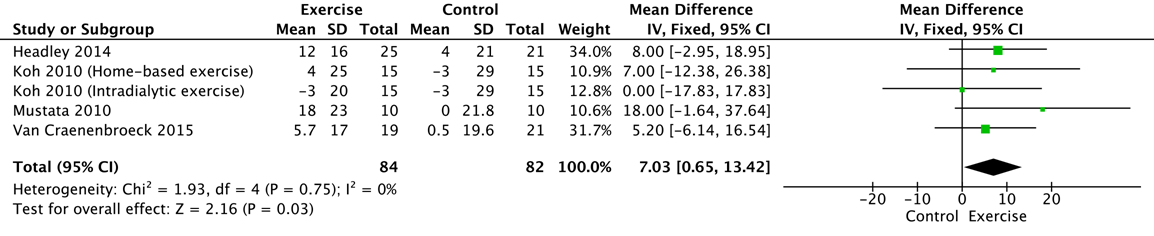
eFigure 6: Forest plot for general health.


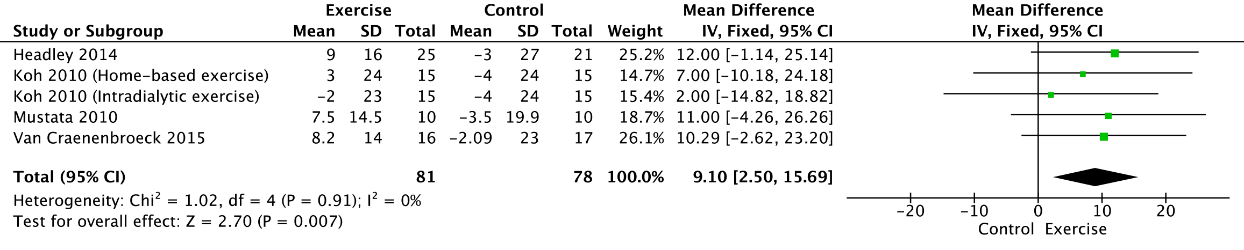
eFigure 7: Forest plot for vitality.

eFigure 8: Subgroup analysis for PWV based on duration of exercise training


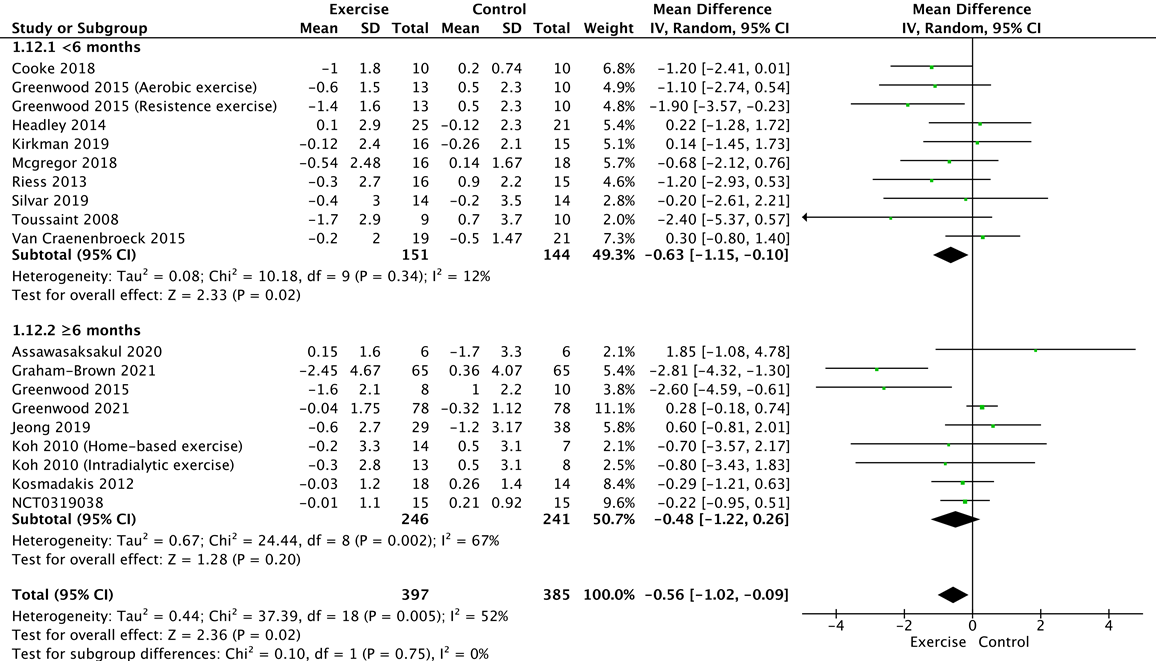

Supplement: Supplementary file 1 [file Data_Sheet_1.docx]
